# Preparation and Properties of Low-Dielectric Polyimide Films Containing Tert-Butyl

**DOI:** 10.3390/polym16070984

**Published:** 2024-04-04

**Authors:** Xin Li, Rongrong Zheng, Cheng Wang, Haiyang Chang, Shuwu Chen, Liyan Wang, Xue Cui, Yutao Liu, Junhao Li, Guangning Yu, Ji Shi

**Affiliations:** 1 School of Petrochemical Engineering, Shenyang University of Technology, Liaoyang 111003, China; 18366036661@163.com (X.L.); wangliyan0122@163.com (L.W.); cheetos1030@163.com (X.C.); liuyutao328@163.com (Y.L.); leejwh@163.com (J.L.); y18340984398@163.com (G.Y.); 18641971205@163.com (J.S.); 2 School of Chemical Engineering and Light Industry, Guangdong University of Technology, Guangzhou 510006, China; changhy0922@163.com; 3 Guangdong Provincial Laboratory of Chemistry and Fine Chemical Engineering Jieyang Center, Jieyang 522000, China; 4 Aromatics Laboratory, Liaoyang Petrochemical Company, Liaoyang 111003, China; chenshuwu@petrochina.com

**Keywords:** low-dielectric, polyimides, tert-butyl, microelectronics industry, films

## Abstract

The design of high-performance polyimide (PI) films and understanding the relationship of the structure–dielectric property are of great significance in the field of the microelectronics industry, but are challenging. Herein, we describe the first work to construct a series of novel tert-butyl PI films (denoted as PI-1, PI-2, PI-3, and PI-4) based on a low-temperature polymerization strategy, which employed tetracarboxylic dianhydride (pyromellitic anhydride, 3,3′,4,4′-biphenyl tetracarboxylic anhydride, 4,4′-diphenyl ether dianhydride, and 3,3′,4,4′-benzophenone tetracarboxylic anhydride) and 4,4′-diamino-3,5-ditert butyl biphenyl ether as monomers. The results indicate that introducing tert-butyl branches in the main chain of PIs can enhance the free volume of the molecular chain and reduce the interaction between molecular chains of PI, resulting in a low dielectric constant. Particularly, the optimized PI-4 exhibits an excellent comprehensive performance with a high (5) wt% loss temperature (454 °C), tensile strength (117.40 MPa), and maximum hydrophobic angle (80.16°), and a low dielectric constant (2.90), which outperforms most of the results reported to date.

## 1. Introduction

With the rapid development of the microelectronics industry, the requirements for low-dielectric and low-energy-consumption devices are increasing, wherein interlayer dielectric materials are crucial in electronic devices [1,2,3,4,5]. In this context, low-dielectric materials have attracted much interest. Particularly, polyimide (PI) films are considered as one of the most promising candidates for the next generation of dielectrics due to their excellent thermal [6,7,8], mechanical [9,10], and dielectric properties [11,12,13,14,15], and have been applied in insulation layers, buffer coatings, and passivation layers in the microelectronics industry [16,17,18,19]. However, it is a fact that the dielectric constant of most PI films is not low enough (approximately 3.2–4.0). Therefore, it is urgent to further reduce the dielectric constant of PI and improve its various properties. Although great efforts have been made, most of the reported PI films have unsatisfying heat resistance, mechanical properties, and/or dielectric constant, to some extent. Therefore, it is highly desired that we design high-performance PI materials.

As for the synthesis of PI films, tetracarboxylic anhydride and aromatic diamine are commonly used as monomers for ring-opening addition reactions. During this process, the structure of the monomers has a significant impact on the properties of PI films [20,21,22,23,24]. Particularly, modifying the side or main chain of PI can effectively alter the dielectric constant [25,26]. For example, Aldred et al. [27] found that increasing the number of benzene rings in the side chains of aromatic diamine could reduce the dielectric constant of PI films. In addition, Zuo and co-workers [28] developed a new strategy to construct low-dielectric-constant PI films by the introduction of meta-substituted and trifluoromethyl structures into the main chains of PI films, in which the meta-substituted structure and trifluoromethyl units could inhibit the transfer of charge transfer complex effects and increase the free volume of PI films, thereby resulting in a lower dielectric constant. Recently, Wu et al. [29] reported three fluorene-based PI films with silyl ether structures that delivered a low dielectric constant and high hydrophobicity. The reason is that the bulky nonpolar tert-butyldiphenylsilyl side groups in the PI films are able to decrease the charge transfer interaction and electronic conjugation along the PI chain, and increase the free volume and hydrophobicity of Si-PI films. Additionally, Lu et al. [30] found that increasing the tight packing of the molecular chain and the strong intermolecular interaction force could improve the thermal stability and mechanical properties of PI films. Inspired by these achievements and our interest in high-performance PI films, there is great potential to design high-performance PI films through rational molecular structure design. However, the relationship of the structure–dielectric property of PI is still ambiguous and barely studied.

In this work, four novel tert-butyl PI films (denoted as PI-1, PI-2, PI-3, and PI-4) with different anhydride structures in the main chain were successfully synthesized through a low-temperature polymerization strategy. Various measurements were performed to investigate the properties of the synthesized PI films, and the relationship of the molecular structure and the dielectric properties of PI films was studied in detail. The results indicate that introducing a tert-butyl side chain in the PI structure can enhance the free volume of the molecular chain and reduce the interaction between the molecular chain of PI, resulting in a low dielectric constant. In particular, the well-designed PI-4 delivers a high (5) wt% loss temperature of 454 °C, tensile strength of 117.399 MPa, and maximum hydrophobic angle of 80.16°, and a low dielectric constant of 2.9, which is superior to most of results reported to date (Table S1). This work provides an approach of adjusting the type of main chain of PIs to achieve a lower dielectric constant without sacrificing other properties, and has great potential to be extended to the design of other PI films. Meanwhile, this work offers a simple method for preparing high-performance PI films with excellent comprehensive properties, giving them potential applications in the area of the microelectronics industry.

## 2. Experiment Section

### 2.1. Materials

Potassium carbonate was purchased from Jinan Luhui Chemical Co., Ltd., Jinan, China. Anhydrous methanol, dichloromethane, and triethylamine were purchased from Chengdu Kelong Chemical Co., Ltd., Chengdu, China. p-Chloronitrobenzen, ethanol, pyromellitic dianhydride(PMDA), palladium on carbon (10% Pd/C), ethyl acetate, 3,3′,4,4′-biphenyl tetracarboxylic anhydride (BPDA), 4,4′-diphenyl ether dianhydride (ODPA), N,N-dimethylformamide, 3,3′,4,4′-benzophenone tetracarboxylic anhydride (BTDA), 1-methyl-2-pyrrolidinone, and 2,6-di-tert-butyl-4-nitrophenol were purchased from Shanghai Macklin Biochemical Technology Co., Ltd., Shanghai, China. Sodium chloride solution was supplied by Tianjin Beilian Fine Chemicals Development Co., Ltd., Tianjin, China. 1,4-Dioxane and 4,4-diaminodiphenyl ether were purchased from Shanghai Aladdin Biochemical Technology Co., Ltd., Shanghai, China. Ammonium formate was obtained from Jinan Kaichuang Chemical Co., Ltd., Jinan, China. All reagents were used as received without further purification.

### 2.2. Characterization

Nuclear magnetic resonance (NMR) data were obtained using a Bruker Model AVANCE III 400 spectrometer (Brookbyesbing, Switzerland). Fourier-transform infrared (FT-IR) spectra were recorded on a Bruker Tensor 27 spectrometer (Brookbyesbing, Switzerland). Thermogravimetric analyses (TGAs) were performed using a TG209F1 libra thermal analyzer (Selb, Bavaria, Germany) with a heating rate of 10 °C min^−1^ from 25 to 800 °C under nitrogen. The dynamic mechanical spectra were obtained using DMA Q800 analyzer (New Castle, DE, USA) in tensile mode at a preload force of 0.01 N, an amplitude of 20 mm, and a force track of 125% at a frequency of 1 Hz and a heating rate of 5 °C min^−1^. Thermal mechanical analysis (TMA) was determined by TMA Q400 analyzer (New Castle, DE, USA) at a preload force of 0.05 N and a heating rate of 10 °C min^−1^ from 50 to 400 °C. The dielectric constant k value was measured by Solartron SI 1260 impedance/gain phase analyzer at frequencies from 100 Hz and 1 Hz at 25 °C and 40% relative humidity. Field emission scanning electron microscope (SEM) images were supplied by Hitachi S-4800 (Tokyo, Japan).

### 2.3. Synthesis of 4,4′-Diamino-3,5-Ditert Butyl Biphenyl Ether

**Scheme 1:**
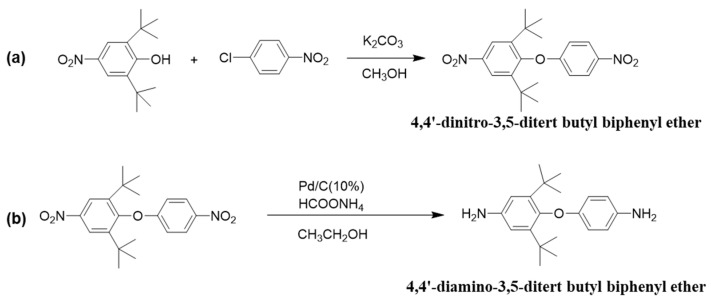
Synthesis of 4,4′-diamino-3,5-ditert butyl biphenyl ether. synthesis of 4,4′-dinitro-3,5-ditert butyl biphenyl ether (**a**) and 4,4′-diamino-3,5-ditert butyl biphenyl ether (**b**).

The synthetic procedure of 4,4′-diamino-3,5-ditert butyl biphenyl ether is shown in Scheme 1. The synthesis of 4,4′-diamino-3,5-ditert butyl biphenyl ether consists of two steps. Step a: p-Chloronitrobenzene (0.76 g, 4.80 mmol), potassium carbonate (0.08 g, 0.58 mmol), and anhydrous methanol (5.00 mL) were added to a 25 mL three-necked flask under magnetic stirring and nitrogen protection conditions. Then, the mixture was transferred to a low-temperature tank (0 °C). Afterwards, the solution of 2,6-di-tert-butyl-4-nitrophenol (0.50 g, 19.89 mmol) in dichloromethane (5.00 mL) was added dropwise to the above mixture. The reaction solution was further heated to 80 °C and refluxed for 24 h. After reaction, the solution was cooled to room temperature. Then, the solution was transferred to a saturated sodium chloride ice water solution. Subsequently, the crude 4,4′-dinitro-3,5-ditert butyl biphenyl ether crystal was precipitated and filtered. To obtain a high-purity product, the crude 4,4′-dinitro-3,5-ditert butyl biphenyl ether crystal was subject to a recrystallization treatment using methanol. The yield of 4,4′-dinitro-3,5-ditert butyl biphenyl ether was approximately 80.50%.

Step b: The prepared 4,4′-dinitro-3,5-ditert butyl biphenyl ether (0.50 g, 1.34 mmol) and Pd/C catalyst (23.00 mg, 0.22 mmol) were added to a 25.00 mL three-necked flask, accompanied by stirring and nitrogen protection. After that, 1,4-dioxane (5.00 mL) was introduced into the mixture to obtain a suspension. Then, the mixture was heated to 80 °C and refluxed. Subsequently, ethanol solution (5.00 mL) containing 25.00 wt% of ammonium formate was slowly added to the mixture, followed by refluxing for 24 h. After the reaction ends, the reaction solution was filtered to remove Pd/C catalyst. Subsequently, the filtrate was transferred into the saturated sodium chloride ice water solution. After drying, the column chromatography was employed using dichloromethane and ethyl acetate (*v*/*v* = 1:1) as eluents to obtain the 4,4′-diamino-3,5-ditert butyl biphenyl ether monomer. The yield of 4,4′-diamino-3,5-ditert butyl biphenyl ether was approximately 82.30%.

4,4′-diamino-3,5-ditert butyl biphenyl ether (white powder, yield: 82.30%). ^1^H NMR (400 MHz, DMSO-*d*_6_, δ, ppm): 8.20 (d, 1H, H^10^), 8.18 (d, 1H, H^9^), 7.80 (d, 1H, H^13^), 7.65 (s, 1H, H^5^), 7.50 (s, 1H, H^1^), 7.31 (d, 1H, H^12^), 3.25 (s, 2H, H^14^), 2.51 (s, 1H, H^15^), and 1.41 (s, 3H, H^17+18+19+21+22+23^).

### 2.4. Preparation of Tert-Butyl PI Films

**Scheme 2:**
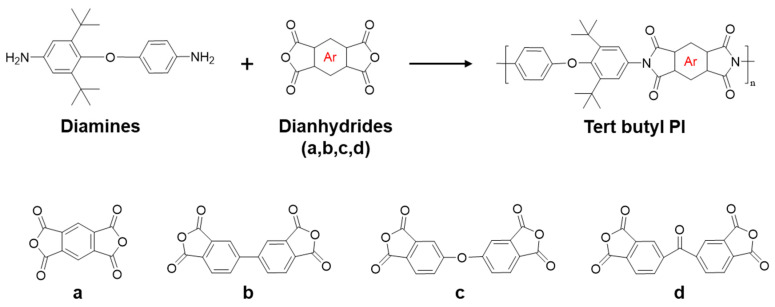
Synthesis of tert-butyl PI films. (**a**) PMDA (**b**) BPDA (**c**) ODPA (**d**) BTDA.

The preparation of tert-butyl PI films consists of two stages (Scheme 2). First, 4,4′-diamino-3,5-ditert butyl biphenyl ether (6.24 g, 0.02 mol) was dissolved into the 1-methyl-2-pyrrolidinone (NMP) (35.00 mL), and then the mixture was placed in a low-temperature bath (10 °C). Afterwards, pyromellitic dianhydride (PMDA) (4.36 g, 0.02 mol) dissolved into a small amount of NMP was introduced into the mixture solution to achieve a complete dissolution. After 8 h, a viscous yellow polyamide acid (PAA) solution with a solid content of 20.00% was obtained. Second, the viscous PAA solution was poured into a clean T-shaped mold and dried at a vacuum oven for 24 h to remove the bubbles of PAA solution. Subsequently, the temperature of vacuum oven was gradually raised to 80 °C and dried for 10 h to obtain a yellow PI film (denoted as PI-1). The preparation of other PI films (PI-2, PI-3, and PI-4) is similar to that of PI-1, except that 3,3′,4,4′-biphenyl tetracarboxylic anhydride (BPDA), 4,4′-diphenyl ether dianhydride (ODPA), and 3,3′,4,4′-benzophenone tetracarboxylic anhydride (BTDA) were employed as monomers, respectively. These free-standing films are shown in Figure S1.

**PIs** (yellow powder, yield: 92.50%). FT-IR (KBr, cm^−1^): 2932, 1670, 1386, and 657.

### 2.5. Preparation of Traditional PI Film without Tert-Butyl Group

**Scheme 3:**
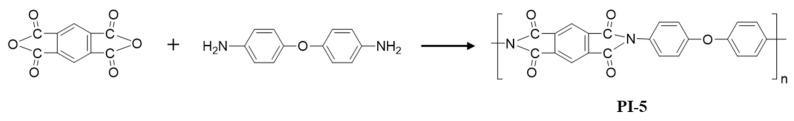
Synthesis of traditional PI film (PI-5) without tert-butyl group.

To compare the performance with tert-butyl PI films, a traditional PI film without tert-butyl group was selected as a control sample. The structure of traditional PI film is shown in Figure S2. The synthetic procedure is as follows (Scheme 3). First, 4,4-diaminodiphenyl ether (2.05 g, 15.00 mmol) was dissolved into N, N-dimethylformamide (DMF, 20.00 mL) at 10 °C and nitrogen atmosphere. Then, terephthalic anhydride (3.27 g, 15.00 mmol) was added to the mixture solution. After reaction for 8 h at 180 °C, a viscous yellow PAA solution was obtained. Subsequently, the viscous yellow PAA solution was transferred into a PTFE mold and dried at a vacuum oven for 24 h. Finally, the traditional PI film was formed (denoted as PI-5).

**PIs** (yellow films, yield: 88.90%). FT-IR (KBr, cm^−1^): 2932, 1670, 1386, and 657.

### 2.6. Density Functional Theory (DFT) Calculations

Density functional theory (DFT) calculations were carried out using Materials Studio 8.0. Eight different PI fragments were selected as model molecules. Their electronic structures at ground state condition were analyzed by DFT. Geometric optimization was performed using a DMol3 module, and was obtained at potential energy minima. The molecular orbital energy levels of optimal PI configuration were calculated using a mixed functional at the B3LYP level (combining Hartree Fock and DFT) to determine the highest occupied molecular orbital (HOMO) and lowest unoccupied molecular orbital (LUMO). The dielectric constant of those optimal configurations was calculated using the molecular dynamic simulation (MD) method. The nonpolarizable COMPASS II force field with the forcite module in the Material Studio simulation package was employed. The static dielectric constant is based on the dipole moment fluctuation without an external electric field.

## 3. Results and Discussion

### 3.1. Structure Characterization of 4,4′-Diamino-3,5-Ditert Butyl Biphenyl Ether

**Figure 1:**
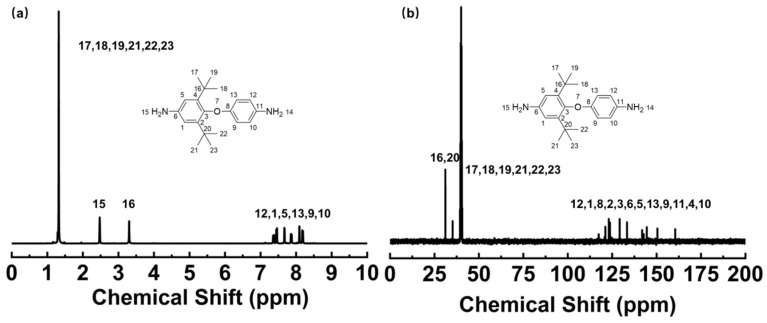
NMR characterization of 4,4′-diamino-3,5-ditert butyl biphenyl ether: (**a**) ^1^H NMR; and (**b**) ^13^C NMR.

The structure of the prepared 4,4′-diamino-3,5-ditert butyl biphenyl ether was first characterized by the nuclear magnetic resonance hydrogen spectrum (^1^H NMR). As shown in Figure 1a, the chemical shift at 1.4 ppm is attributed to the -CH_3_ group of 4,4′-diamino-3,5-ditert butyl biphenyl ether. The chemical shifts at 2.5 ppm and 3.30 ppm are assigned to the -NH_3_ group on both sides of the 4,4′-diamino-3,5-ditert butyl biphenyl ether. The chemical shifts at 7–8.5 ppm correspond to the benzene ring. After that, the nuclear magnetic resonance carbon spectrum (^13^C NMR) was employed to obtain further information. As shown in Figure 1b, the chemical shifts at 31.6 ppm, 34.7 ppm, and 39 ppm represent the carbon signals of tert-butyl in the diamine monomer. The chemical shifts at 110 ppm–155 ppm are the carbon signals of the benzene ring of 4,4′-diamino-3,5-ditert butyl biphenyl. Therefore, it can be concluded that 4,4′-diamino-3,5-ditert butyl biphenyl ether is successfully synthesized.

### 3.2. Structure Characterization of Tert-Butyl PI Films

**Figure 2:**
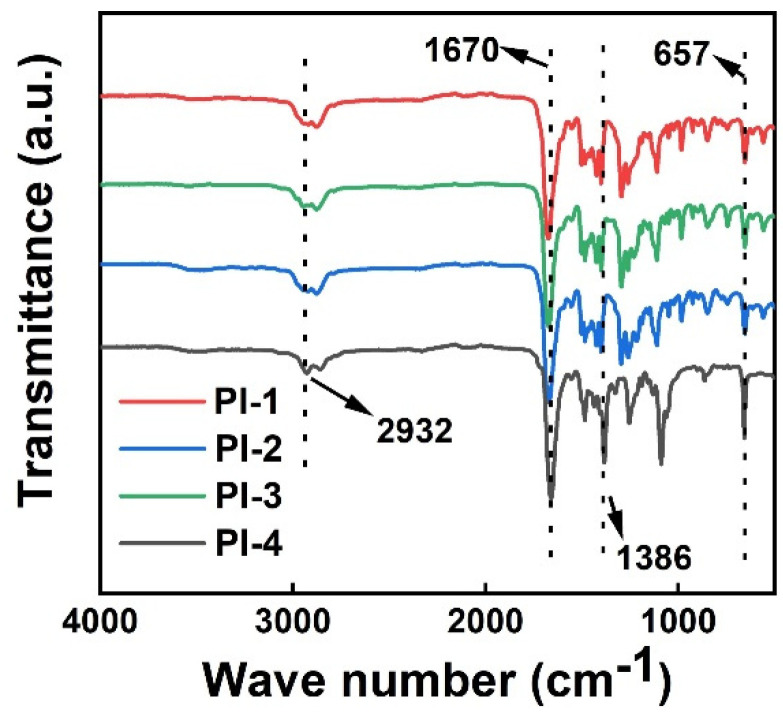
FT−IR spectra of tert-butyl PI films.

The Fourier-transform infrared spectrum (FT-IR) was employed to study the synthesized tert-butyl PI films. As shown in Figure 2, it can be observed that the four tert-butyl PI films (PI-1, PI-2, PI-3, and PI-4) show similar characteristic absorption peaks. In particular, the peak at 657 cm^−1^ represents the bending vibration of the C=O group in tert-butyl PI films, and the peak at 1386 cm^−1^ is attributed to the C-N stretching vibration of acyl amide group. The peak at 2932 cm^−1^ is assigned to the C-H vibration of tert-butyl in the imide structure. The peak at 1670 cm^−1^ is the carbonyl absorption peak in the imide. The above results indicated that the tert-butyl group was successfully introduced into the PI films.

**Figure 3:**
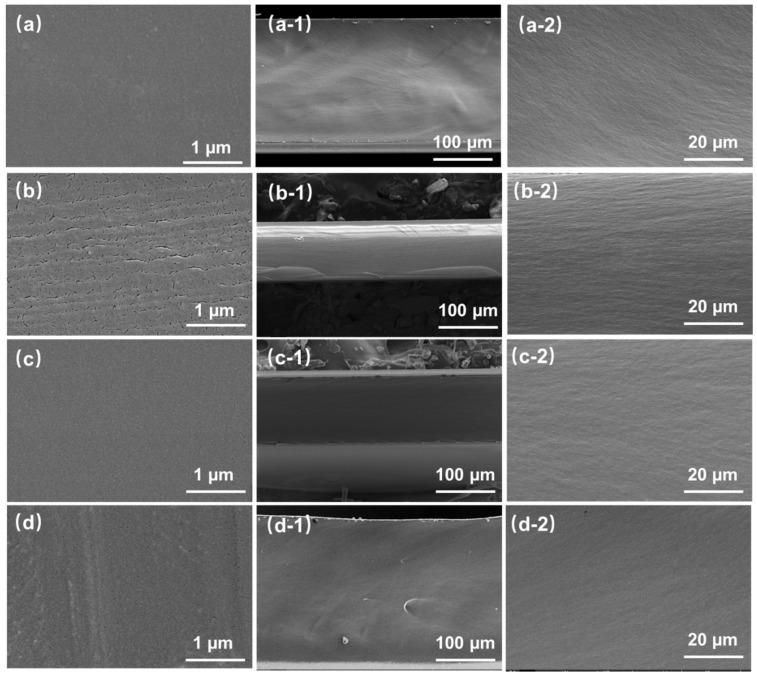
SEM images of tert-butyl PI films: (**a**) PI-1; (**b**) PI-2; (**c**) PI-3; and (**d**) PI-4. (Note: (**a-1**,**a-2**,**b-1**,**b-2**,**c-1**,**c-2**,**d-1**,**d-2**) are the cross-sectional images of tert-butyl PI films).

Subsequently, scanning electron microscopy (SEM) was used to investigate the surface structure of tert-butyl PI films (Figure 3). It can be observed that PI-1 and PI-3 show a flat and smooth surface, while PI-2 and PI-4 display a rough surface structure (Figure 3b,d), wherein PI-2 prepared using BPDA as a monomer exhibits cotton-like grooves. Figure 3(b-1,b-2) show the electron microscope cross-section of PI-2. It can be found that there is no obvious aggregation in PI-2. Meanwhile, the cross-sectional images of four tert-butyl PI films exhibit relatively smooth cross-sections in Figure 3, while the flocculent substances on both sides of the screenshot may be acyl imine groups that have not fully reacted.

### 3.3. Study of Dielectric Properties of Tert-Butyl PI Films

**Figure 4:**
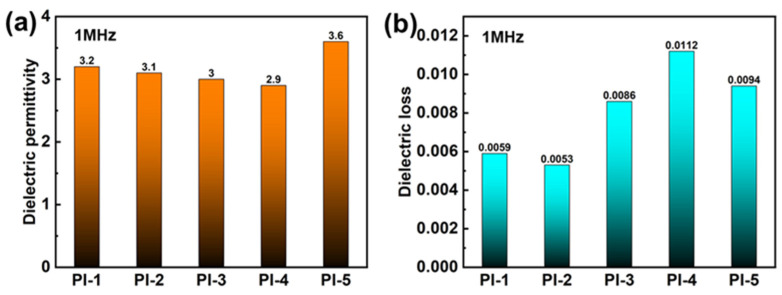
Dielectric properties of tert-butyl PI films: (**a**) dielectric constant; and (**b**) dielectric loss.

The dielectric constant of tert-butyl PI films was studied at room temperature. Figure 4a shows the dielectric constant of tert-butyl PI films at 1 MHz. It can be found that the dielectric constants of PI-1, PI-2, PI-3, and PI-4 at 1 MHz are approximately 3.2, 3.1, 3, and 2.9, respectively. To compare the performance with tert-butyl PI films, a traditional PI film without a tert-butyl group was selected as a control sample. The corresponding structural characterization is shown in Supporting Information (Figures S3 and S4). As shown in Figure 4a, the dielectric constant of PI-5 is approximately 3.6, which is much higher than that of tert-butyl PI films. The structural difference between PI-5 and tert-butyl PI films is focused on the diamine part. The diamine segment of tert-butyl PI is a non-planar tert-butyl structure where the tert-butyl group twists at a certain angle. Such a non-planar conjugated structure in the main chain can hinder the close packing of the polymer chains and segments, which contributes toward increasing the local free volume and reducing the dielectric constant [31,32,33,34]. In addition, the introduction of functional groups (e.g., C=O, -O-, and benzene ring) in anhydride can greatly reduce the molecular polarizability and decrease the packing density, thus resulting in a decrease in the dielectric constant of tert-butyl PI films. In contrast, the PI-5 film contains a diphenyl ether unit, in which the ether linkages in the main chain can make the PI chains easier to form close packing, resulting in a higher dielectric constant. Similar phenomena can be observed in other literatures. In addition, the dielectric loss of PI-1, PI-2, PI-3, PI-4, and PI-5 films at 1 MHz is between 0.005–0.011 (Figure 4b). At the same time, the PI-2 film has a lower dielectric loss, while the PI-4 film exhibits a higher dielectric loss. The reason may be due to the different restrictions on the dipole moment rotation caused by the dense stacking of molecular chains and strong intermolecular interactions [30].

In addition, density functional theory (DFT) calculations [35,36] and molecular dynamics simulations (MD) [30,37,38,39] were employed to further understand the relationship between the structure and dielectric properties of PI. A series of model PI molecules with or without a tert-butyl structure were selected. Since the dielectric constant of PI is related to the distribution of the electron cloud on the HOMO orbital and LUMO orbital [40], we, therefore, compared the HOMO value and LUMO value of these model molecules. As shown in Table 1, Figures S5 and S6, it can be observed that the electron cloud of PI is mainly distributed on the diamine part for the HOMO orbital and moves to the dianhydride part for the LUMO orbital. Meanwhile, it can be found that the HOMO value of PI with a tert-butyl structure is much higher than that of PI without a tert-butyl structure, indicating that introducing a tert-butyl structure in PI is able to affect the electronic cloud distribution on the diamine and increase the energy of HOMO, thereby reducing the dielectric constant.

### 3.4. Study of Thermal Performance of Tert-Butyl PI Films

**Figure 5:**
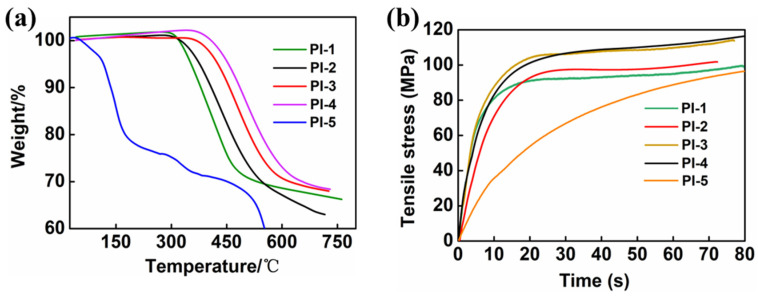
TGA curves (**a**) and stress curves (**b**) of tert-butyl PI films.

As an ideal candidate for 5G high-frequency communications, the thermodynamic stability of PI films has a significant impact on practical applications [41,42,43]. Therefore, the thermal performance of the tert-butyl PI films was also investigated in detail. As shown in Figure 5a, no significant thermal weight loss before 300 °C can be observed while the thermal weight loss of PI-5 begins at approximately 120 °C, indicating that the introduction of a tert-butyl group can enhance the stability of PI films. In addition, the effect of the anhydride structure in tert-butyl PI films on the thermal weight loss was also investigated. As shown in Table 2, the 5% thermal weight loss of PI-1 to PI-4 was 381 °C, 412 °C, 460 °C, and 480 °C, respectively, indicating that the introduction of C=O and ether linkages in anhydride can improve the heat resistance of PI films. When the thermal weight loss is 20%, the decomposition temperature of PI-4 is up to 540 °C. In addition, the traditional PI-5 film without a tert-butyl group was also compared to verify the advantage of tert-butyl PI films (Table 2). When the thermal weight loss reaches 10%, the decomposition temperature of traditional PI-5 films is 135 °C. When the thermal weight loss reaches 20%, the decomposition temperature of traditional PI-5 films is only 175 °C. The thermodynamic performance data fully indicate that introducing a tert-butyl structure in PI films contributes toward enhancing the thermodynamic stability of PI films [44].

### 3.5. Study of Mechanical Performance of Tert-Butyl PI Films

As a promising material for the next-generation ILD, PI films not only require low dielectric constant and excellent thermal properties, but also excellent mechanical properties. Figure 5b shows the variation curves of the tensile stress of tert-butyl PI films over time. It can be found that the tensile strength of tert-butyl PI films is in the range of 90–120 MPa, and the modulus is in the range of 2570–2620 MPa (Table 3). Particularly, PI-4 exhibits a better mechanical performance with a high tensile strength (117.40 MPa) and modulus (2570.01 MPa/mm/mm). In addition, we also studied the tensile strength of traditional PI-5 film. As illustrated in Table 3, the tensile strength (106.29 MPa) and modulus (2517.49 MPa/mm/mm) of the traditional PI-5 film is relatively lower than the PI-4 film. The reason is that introducing tert-butyl on the polyimide chain can enhance the flexibility of the imide structure, and improve the disordered arrangement of the PI films, resulting in an increase in the tensile strength to some extent. In addition, the introduction of C=O and biphenyl groups also increases the rigidity of the structure to a certain extent, which is conducive to the tensile strength. Therefore, both the synergistic effects mentioned above result in an increase in the tensile strength of the synthesized tert-butyl PI films.

### 3.6. Study of Hydrophobic Performance of Tert-Butyl PI Films

**Figure 6:**
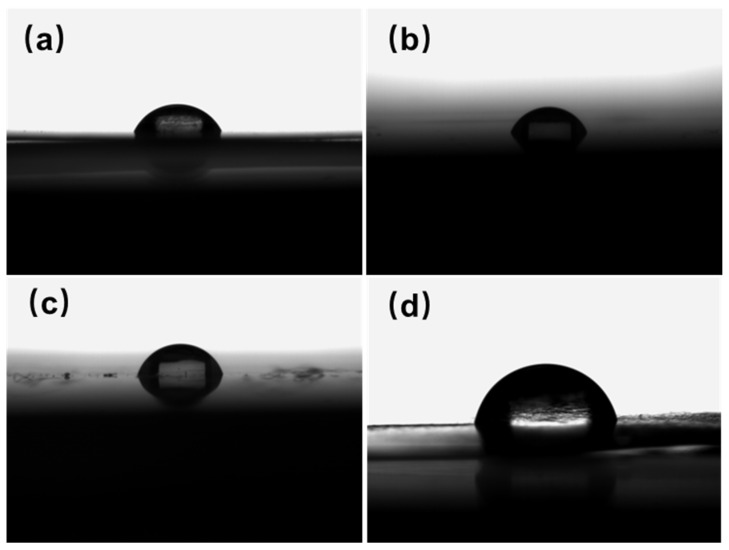
Hydrophobic angle of tert-butyl PI films: (**a**) PI-1; (**b**) PI-2; (**c**) PI-3; and (**d**) PI-4.

The hydrophobicity of tert-butyl PI films was investigated by the water contact angle test. The reason is that water or moisture possesses a significant influence on the dielectric performance of PI films [45,46]. As shown in Figure 6 and Table 4, the contact angle on the surface of PI-1, PI-2, PI-3, and PI-4 PI films increases with the increase in the volume of the anhydride monomer, indicating the increasing hydrophobicity. In particular, the PI-4 film exhibits a maximum hydrophobic angle of 80.16°. The reason is that the large volume of the side chain in tert-butyl PI films reduces the interaction and packing density between molecular chains, which is conducive to improving the hydrophobicity of tert-butyl PI films [47]. Therefore, increasing the volume of anhydride of tert-butyl PI films contributes to reducing the adsorption of water. The decrease in the water absorption property also helps to decrease the dielectric constant, which further explains the low dielectric constant of PI-4. In addition, a control experiment of the traditional PI-5 film without a tert-butyl group was employed (Figure S7 and Table 4). The maximum water contact angle of the traditional PI-5 film is 34.13°, which is significantly lower than tert-butyl PI films, indicating that introducing a tert-butyl group in aromatic diamine can enhance the hydrophobicity of PI films.

## 4. Conclusions

In summary, a series of novel tert-butyl PI films were synthesized for the first time based on a low-temperature polymerization strategy. The introduction of a tert-butyl structure in PI films is beneficial for enhancing the free volume of the molecular chains of PI and weakening the interaction between molecular chains of PI, thus leading to a low dielectric constant and dielectric loss. In particular, the optimized PI-4 exhibits an outstanding comprehensive performance with a high (5) wt% loss temperature of 454 °C, tensile strength of 117.40 MPa, maximum hydrophobic angle of 80.16°, and low dielectric constant of 2.90, outperforming most of the results reported to date. This work opens a new avenue for designing high-performance PI films by adjusting the type of main chain of PI films to achieve a lower dielectric constant without sacrificing other properties, and has great potential to be extended to the design of other PI films. Meanwhile, the excellent comprehensive properties of the prepared tert-butyl PI films give them potential applications in the area of the microelectronics industry.

## Figures and Tables

**Table 1:** DFT results of model PI with or without tert-butyl structure.

Polymer Code	PI-1	PI-2	PI-3	PI-4	PI-1 *	PI-2 *	PI-3 *	PI-4 *
E_HOMO_ (eV)	−4.696	−4.542	−4.562	−4.647	−4.88	−4.764	−4.661	−4.701
E_LOMO_ (eV)	−3.825	−3.466	−3.292	−3.717	−3.895	−3.48	−3.366	−3.702
Dielectric constant	4.12	3.6	3.42	3.13	6.24	5.69	5.03	4.64

Notes: * represents the PI films without tert-butyl structure.

**Table 2:** Thermal performance of tert-butyl PI films.

Polymer Code	Thermal Weight Loss ^a^		
	5%	10%	20%
PI-1	354	381	428
PI-2	376	412	463
PI-3	423	460	512
PI-4	454	480	542
PI-5	117	135	175

^a^ TGA test was conducted under nitrogen atmosphere at a heating rate of 10 °C/min.

**Table 3:** Mechanical performance of tert-butyl PI films.

Polymer Code	Tensile Stress (MPa)	Modulus (MPa/mm/mm)
PI-1	99.70	2619.68
PI-2	102.00	2589.72
PI-3	114.26	2576.72
PI-4	117.40	2570.01
PI-5	106.29	2517.49

**Table 4:** Hydrophobic angle results of PI films.

Polymer Code	Left Contact Angle	Right Contact Angle
PI-1	74.67	74.29
PI-2	75.26	75.18
PI-3	79.12	78.66
PI-4	79.57	80.16
PI-5	34.13	33.96

## Data Availability

Data are contained within the article.

## Supplementary Materials

The following supporting information can be downloaded at: https://www.mdpi.com/article/10.3390/polym16070984/s1, Figure S1: The picture of tert-butyl PI films. (a) PI-1. (b) PI-2. (c) PI-3. (d) PI-4. Figure S2: The chemical structure of PI-5 film. Figure S3: FT−IR spectrum of PI-5 film. Figure S4: SEM image (a) and cross-sectional view (b) of PI-5 film. Figure S5: HOMO-LUMO orbitals of PI model molecules containing tert butyl group. Figure S6: HOMO-LUMO orbitals of PI model molecules without tert butyl group. Figure S7: Water contact angle of PI-5 film; Table S1: Performance comparison of PI film. References [33,48,49,50,51,52] are cited in the supplementary materials
