# Supplementary material for: Preparation and Properties of Low-Dielectric Polyimide Films Containing Tert-Butyl
Source: Polymers (Basel). 2024 Apr 4;16(7):984. doi: 10.3390/polym16070984 (PMC11014062; doi:10.3390/polym16070984)
Supplement: Supplementary file 1 [file polymers-16-00984-s001.zip › polymers-2909340-supplementary.pdf]

Supporting Information

Preparation and properties of low dielectric polyimide films containing tert-butyl

Supplementary figures

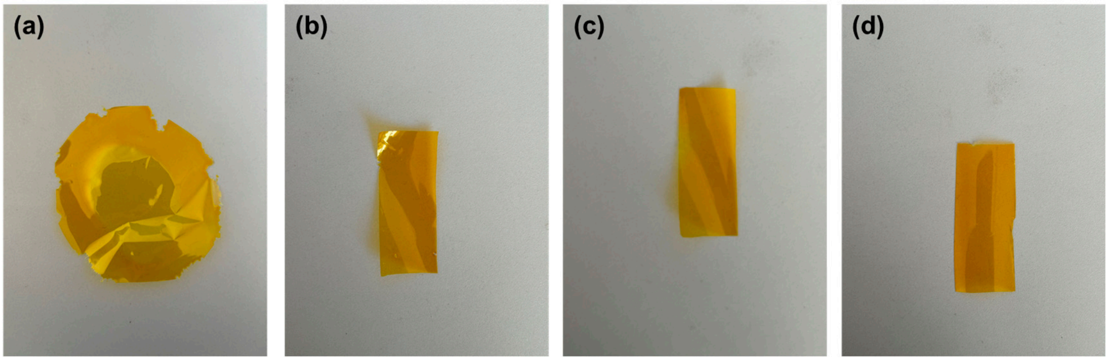

Figure S1. The picture of tert-butyl PI films. (a) PI-1. (b) PI-2. (c) PI-3. (d) PI-4.

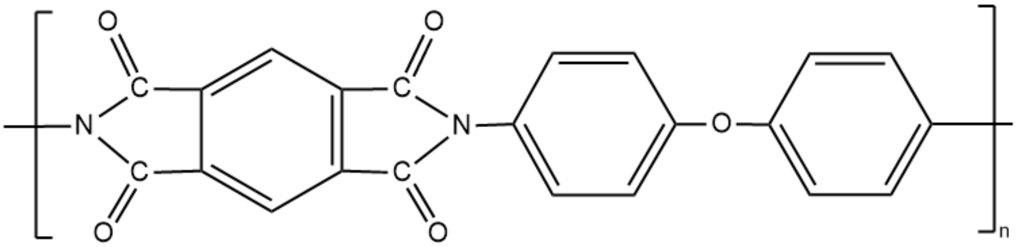

Figure S2. The chemical structure of PI-5 film

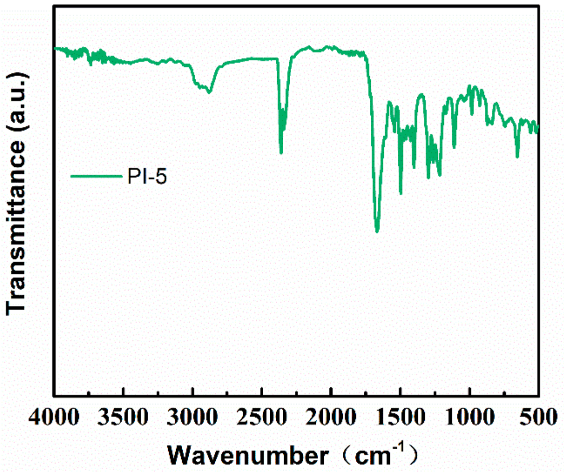

Figure S3. FT-IR spectrum of PI-5 film

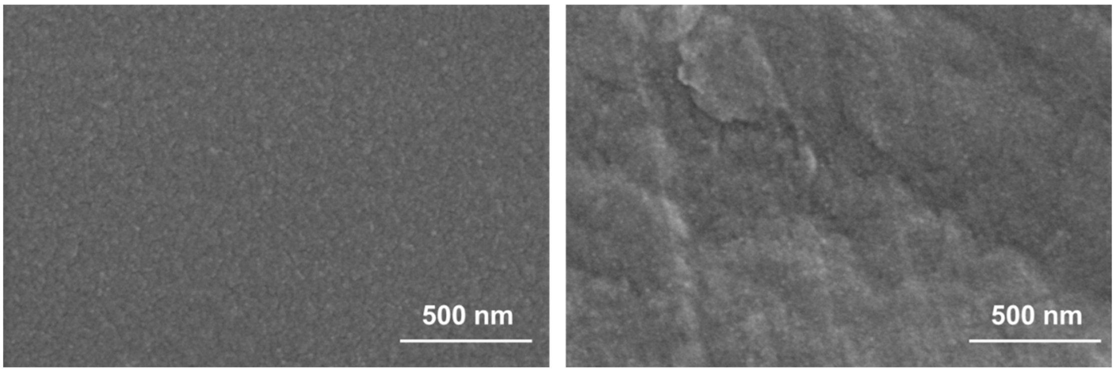

Figure S4. SEM image (a) and cross-sectional view (b) of PI-5 film

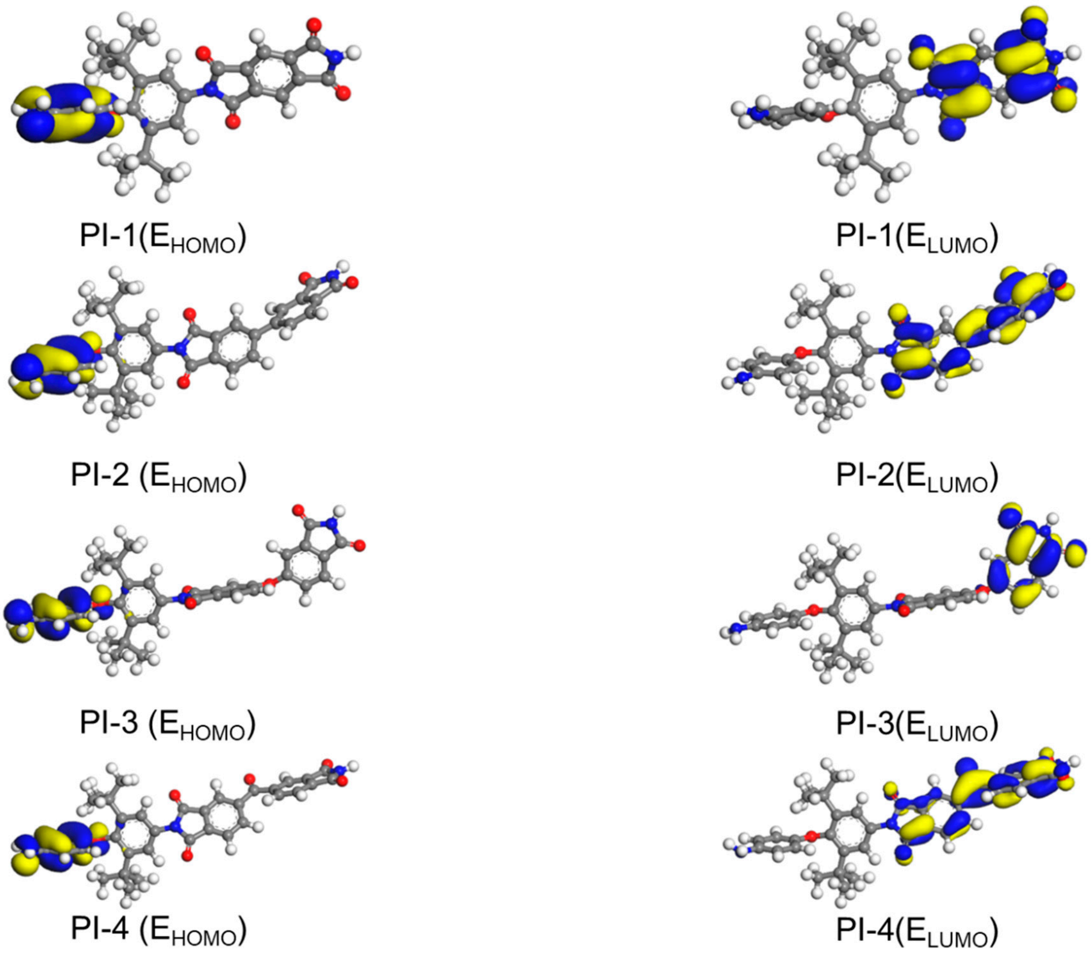

Figure S5. HOMO-LUMO orbitals of PI model molecules containing tert butyl group.

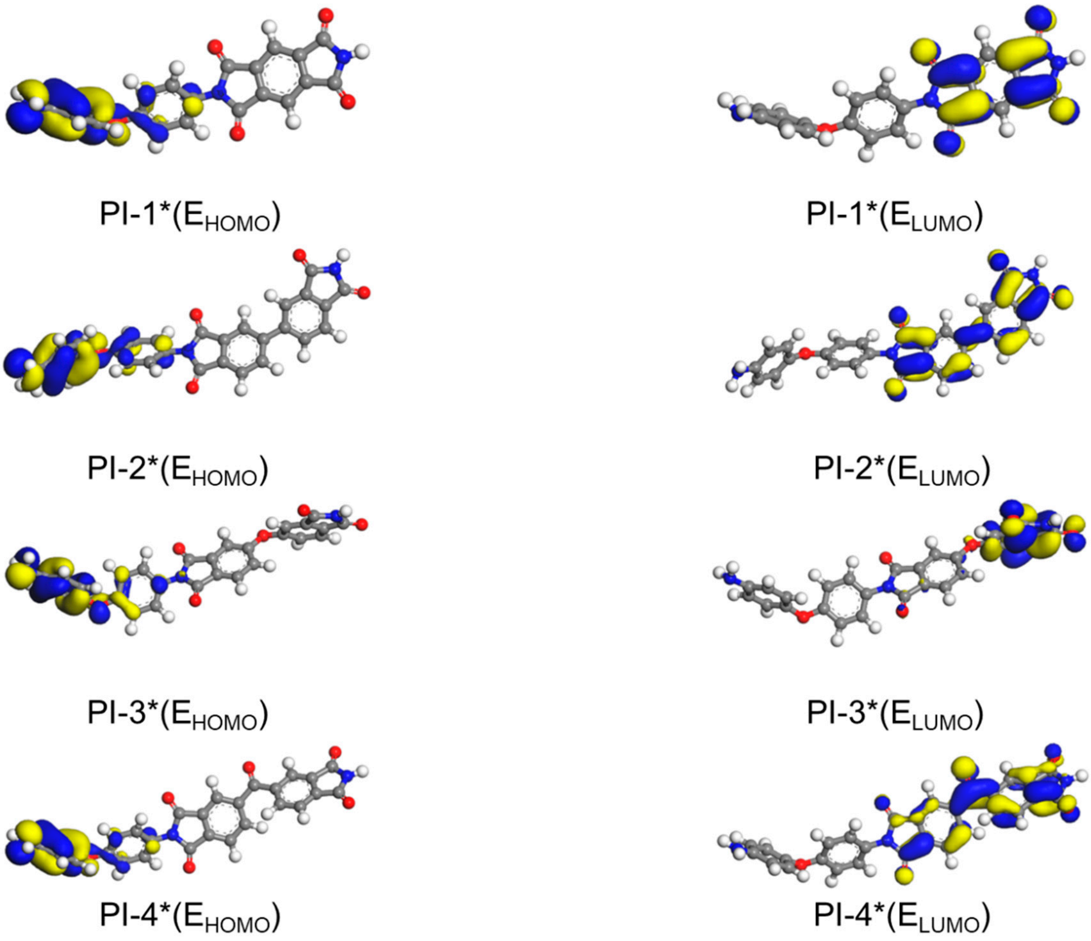

Figure S6. HOMO-LUMO orbitals of PI model molecules without tert butyl group

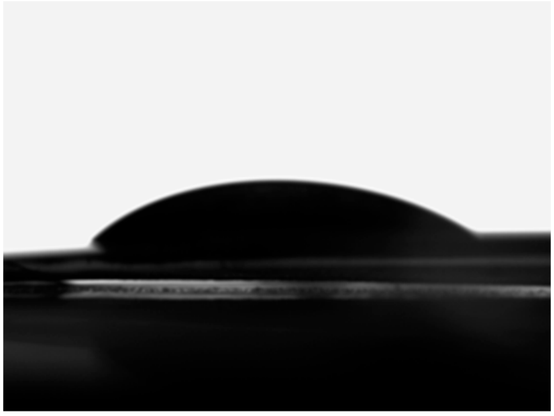

Figure S7. Water contact angle of PI-5 film

Supplementary tables

Table S1. Performance comparison of PI film.

Polymer code	Dielectric constant	5 wt% loss temperature (ºC)	Tensile strength (MPa)	Maximum hydrophobic angle (º)	Reference
BPDA/PDA/TFDB[1]	3.42	--	217.13	--	Composition design and properties investigation of BPDA/PDA/TFDB co-polyimide films with low dielectric permittivity
PI/GNR[2]	3.1	--	166.7	79	Dielectric and mechanical properties of polyimide composite films reinforced with graphene nanoribbon
TiO_2/PI three-layer composite[3]	3.68	--	118	--	Dielectric and mechanical properties of TiO_2/polyimide composites with low dielectric constant
0 wt% h-BN- GNP/PI[4]	3.96	564.9	48.53	--	Enhanced thermal conductivity of

					polyimide composite film filled with hybrid fillers
PI/2wt%BNNS/ANFs[5]	3.73	527.1	110.60	--	Improved mechanical, thermal properties and ideal dielectric properties of polyimide composite films by incorporation of boron nitride nanosheets and aramid nanofibers
BPDA/PDA/TAPOB-PI-0.1[6]	3.12	--	362.2	--	Breaking the mutual restraint between low permittivity and low thermal expansion in polyimide films via a branched crosslink structure
The optimized tert-butyl PI-4	2.9	454	117.399	80.16	This work

Notes: -- means that the data is unavailable.

Reference

[1] X.L. Li, H.Y. Lei, J.C. Guo, J.H. Wang, S.L. Qi, G.F. Tian, D.Z. Wu, Composition design and properties investigation of BPDA/PDA/TFDB co-polyimide films with low dielectric permittivity, Journal of Applied Polymer Science, 136 (2019).

[2] X. Liu, Y. Li, W. Guo, X. Sun, Y. Feng, D. Sun, Y. Liu, K. Yan, Z. Wu, B. Su, J. Yin, Dielectric and mechanical properties of polyimide composite films reinforced with graphene nanoribbon, Surface and Coatings Technology, 320 (2017) 497-502.
[3] G. Zhao, X. Mu, D. Ma, S. Wang, J. Pan, J. Cui, M. Qi, Dielectric and mechanical properties of TiO2/polyimide composites with low dielectric constant, Polymer Engineering & Science, 63 (2023) 1953-1960.
[4] R.Y. Li, C.C. Ding, J. Yu, X.D. Wang, P. Huang, Enhanced thermal conductivity of polyimide composite film filled with hybrid fillers, High Performance Polymers, 33 (2021) 905-913.
[5] T. Zhang, Y.W. Huang, Y. Sun, P.P. Tang, C.Y. Hu, Improved mechanical, thermal properties and ideal dielectric properties of polyimide composite films by incorporation of boron nitride nanosheets and aramid nanofibers, Polymers for Advanced Technologies, 33 (2022) 2123-2136.
[6] H. Zhou, H. Lei, J. Wang, S. Qi, G. Tian, D. Wu, Breaking the mutual restraint between low permittivity and low thermal expansion in polyimide films via a branched crosslink structure, Polymer, 162 (2019) 116-120.
